# Triglyceride-glucose index predicts adverse cardiovascular events in patients with diabetes and acute coronary syndrome

**DOI:** 10.1186/s12933-020-01054-z

**Published:** 2020-06-13

**Authors:** Le Wang, Hong-liang Cong, Jing-xia Zhang, Yue-cheng Hu, Ao Wei, Ying-yi Zhang, Hua Yang, Li-bin Ren, Wei Qi, Wen-yu Li, Rui Zhang, Jing-han Xu

**Affiliations:** Department of Cardiology, Tianjin Chest Hospital, 261 Tai’erzhuang Road, Jinnan District, Tianjin, 300222 China

**Keywords:** Triglyceride-glucose index, Cardiovascular events, Diabetes, Acute coronary syndrome

## Abstract

**Background:**

The triglyceride-glucose index (TyG index) has been regarded as a reliable alternative marker of insulin resistance and an independent predictor of cardiovascular outcomes. Whether the TyG index predicts adverse cardiovascular events in patients with diabetes and acute coronary syndrome (ACS) remains uncertain. The aim of this study was to investigate the prognostic value of the TyG index in patients with diabetes and ACS.

**Methods:**

A total of 2531 consecutive patients with diabetes who underwent coronary angiography for ACS were enrolled in this study. Patients were divided into tertiles according to their TyG index. The primary outcomes included the occurrence of major adverse cardiovascular events (MACEs), defined as all-cause death, non-fatal myocardial infarction and non-fatal stroke. The TyG index was calculated as the ln (fasting triglyceride level [mg/dL] × fasting glucose level [mg/dL]/2).

**Results:**

The incidence of MACE increased with TyG index tertiles at a 3-year follow-up. The Kaplan–Meier curves showed significant differences in event-free survival rates among TyG index tertiles (P = 0.005). Multivariate Cox hazards regression analysis revealed that the TyG index was an independent predictor of MACE (95% CI 1.201–1.746; P < 0.001). The optimal TyG index cut-off for predicting MACE was 9.323 (sensitivity 46.0%; specificity 63.6%; area under the curve 0.560; P = 0.001). Furthermore, adding the TyG index to the prognostic model for MACE improved the C-statistic value (P = 0.010), the integrated discrimination improvement value (P = 0.001) and the net reclassification improvement value (P = 0.019).

**Conclusions:**

The TyG index predicts future MACE in patients with diabetes and ACS independently of known cardiovascular risk factors, suggesting that the TyG index may be a useful marker for risk stratification and prognosis in patients with diabetes and ACS.

## Background

Diabetes is one of the major risk factors for coronary artery disease (CAD) [1]. Up to 37% of patients presenting with acute coronary syndrome (ACS) suffer from diabetes mellitus in China [2]. Compared with those without diabetes, patients with diabetes and ACS remain at higher risk for recurrent ischemic cardiovascular events (CVEs) despite optimal treatment according to the current guidelines [2–4]. Therefore, it is crucial to identify patients at a high risk of developing future CVEs so that intense treatment can be provided. The identification of rapidly available and reliable markers may have great clinical significance in optimizing the risk stratification of recurrent cardiovascular risk.

The triglyceride-glucose index (TyG index), which is calculated from fasting glucose and triglycerides, has been proposed as a reliable marker of insulin resistance (IR) in clinical practice [5, 6]. The TyG index showed better performance for assessing IR than the homeostasis model assessment of IR (HOMA-IR) [7, 8]. Several studies have found a positive association between the TyG index and cardiovascular risk, including systematic CAD, carotid atherosclerosis, hypertension, metabolic syndrome, arterial stiffness and coronary artery calcification [9–15]. Furthermore, recent data suggest the TyG index could provide significant prognostic information in patients with established CAD [16–19]. The TyG index is associated with not only the incidence of cardiovascular disease (CVD) but also the development of Type 2 diabetes (T2DM) [6, 20–24]. Taken together, these results suggest that it may be plausible to use the TyG index as a predictor of future cardiovascular risk in patients with diabetes and CAD.

A previous study of patients with diabetes and stable CAD demonstrated that the TyG index was a useful marker for predicting clinical outcomes [19]. Another recent study found that the TyG index may be a valuable predictor of adverse cardiovascular outcomes after percutaneous coronary intervention (PCI) in patients with diabetes and ACS [25]. To date, no relevant study has focused on the impact of the TyG index on MACE in patients with diabetes and ACS who underwent non-invasive or invasive (PCI or CABG) treatment. To address the knowledge gap, this study aimed to specifically investigate whether the TyG index has a prognostic value for major adverse cardiovascular events (MACE) in patients with diabetes and ACS who received different treatments.

## Methods

### Study population

This study was a single-center, retrospective, observational cohort study. From January 2016 to December 2016, 3428 consecutive patients with T2DM and ACS who were admitted to Tianjin Chest Hospital for coronary angiography were enrolled in this study. We included patients with a history of T2DM who were currently using insulin or hypoglycemic medications, or those with a fasting blood glucose (FBG) ≥ 7.0 mmol/L or a 2-h plasma glucose level on their oral glucose tolerance test (OGTT) ≥ 11.1 mmol/L. Patients with diabetic symptoms underwent the OGTT test during this hospitalization. ACS was defined as including either unstable angina pectoris (UAP), non-ST-segment elevation myocardial infarction (NSTEMI), or ST-segment elevation myocardial infarction (STEMI). Those with severe valvular disease or congenital heart disease requiring cardiac surgery (n = 42), acute infection (n = 76), malignancy (n = 14), severe hepatic dysfunction (n = 18), severe kidney dysfunction (n = 172), nutritional derangements (n = 8), or other severe medical illnesses, or those lacking complete clinical data (n = 285) were excluded. A total of 2815 patients participated in the research. Patients were followed up from January 2019 to December 2019 by telephone or outpatient clinical visit, and 2531 (89.9%) patients completed the 3-year clinical follow-up. The patients were divided into tertiles according to their admission TyG index levels: tertile 1 (n = 844, TyG index ≤ 8.848), tertile 2 (n = 843, 8.849 ≤ TyG index ≤ 9.382) and tertile 3 (n = 844, TyG index ≥ 9.383). This study was approved by the local research ethics committee and strictly adhered to the Declaration of Helsinki. Given the retrospective nature of the present research, no informed consent was required.

### Data collection and definitions

Clinical data were collected from all of the medical records by trained clinicians who were blinded to the purpose of the study. The data included age, gender, duration of diabetes, whether diabetes had been newly diagnosed, smoking history, history of hypertension, family history of CAD, previous myocardial infarction (MI), previous percutaneous coronary intervention (PCI), previous coronary artery bypass graft (CABG), previous stroke, height, weight, systolic and diastolic blood pressure (SBP and DBP), heart rate (HR), left ventricle ejection fraction (LVEF) and medication at discharge. Peripheral venous blood samples were collected early in the morning after an overnight fast on admission and analyzed shortly after sampling. Hemoglobin, FBG, hemoglobin A1c (HbA1c), total cholesterol (TC), triglycerides (TG), low-density lipoprotein-C (LDL-C), high-density lipoprotein-C (HDL-C), serum creatinine, serum uric acid, high-sensitivity C-reactive protein (hs-CRP) and N-terminal proB-type natriuretic peptide (NT-proBNP) levels were analyzed. Renal function was assessed using the baseline estimated glomerular filtration rate (eGFR). Body mass index (BMI) was defined as weight (kg)/height (m^2^). All of the patients underwent coronary angiography during this hospitalization. Significant stenosis was defined as ≥ 50% diameter stenosis in at least one major coronary artery and multivessel disease was defined as ≥ 2 vessels with significant stenosis as observed during angiography. The Global Registry of Acute Coronary Events (GRACE) risk score was calculated for each patient according to eight variables on admission, including age, SBP, HR, presence of cardiac arrest during presentation, Killip class, ST-segment deviation, serum creatinine and positive cardiac biomarkers [26]. The TyG index was calculated as the ln (fasting TG level [mg/dL] × FBG level [mg/dL]/2).

### Endpoints

The primary endpoint was new-onset major adverse cardiovascular event (MACE), defined as the composite of all-cause death, non-fatal MI and non-fatal stroke. All-cause death referred to death attributed to cardiovascular or non-cardiovascular causes. The secondary endpoints included all-cause death, non-fatal MI and non-fatal stroke.

### Statistical analysis

Continuous variables were expressed as mean ± standard deviation when normally distributed. The GRACE score, TG, hs-CRP and NT-proBNP were not normally distributed; therefore, those variables were expressed as medians with interquartile ranges. Categorical variables were presented as frequencies. Baseline demographic characteristics, clinical presentation, laboratory findings, extent of CAD, revascularization and medication data were compared between groups by analysis of variance or Kruskal–Wallis tests for continuous variables, and with a Chi square test or Fisher’s exact test for categorical variables. Multivariate linear regression analyses based on the stepwise method were performed to reveal the factors associated with the TyG index. The Kaplan–Meier event-free survival curves associated with TyG index tertiles were compared using log-rank tests. A multivariate stepwise Cox proportional hazards regression analysis with entry/stay criteria of 0.1/0.1 was constructed to identify independent predictors of MACE. The possible factors included age, sex, duration of diabetes, smoking history, hypertension, previous MI, previous PCI, previous CABG, previous stroke, BMI, AMI, LVEF, left main disease, multi-vessel disease, revascularization, HbA1c, LDL-C, uric acid, hs-CRP, NT-proBNP, eGFR, statin use, insulin use and TyG index. The area under the receiver operating characteristic (ROC) curves was used to indicate the predictive value of the TyG index for MACE. To evaluate whether an increased TyG index had incremental predictive value for MACE, C-statistics, net reclassification improvement (NRI) and integrated discrimination improvement (IDI) were compared between models. A two-sided analysis with a *P* value < 0.05 was considered significant. All of the analyses were performed using SPSS version 20.0 (IBM Corp, Armonk, NY, USA) and SAS version 9.1.3 (Cary, NC, USA).

## Results

### Baseline characteristics of patients

Baseline clinical characteristics and clinical events data were fully recorded for 2531 patients (89.9%). Patient characteristics are listed in Table 1. The study patients had an average age of 66.3 ± 6.8 years and 1415 (55.9%) patients were male. Patients were divided into tertiles according to the admission TyG index levels (tertile 1: n = 844, TyG index ≤ 8.848; tertile 2: n = 843, 8.849 ≤ TyG index ≤ 9.382; and tertile 3: n = 844, TyG index ≥ 9.383). The mean levels of TyG index of the three groups were 8.467 ± 0.293, 9.114 ± 0.152 and 9.841 ± 0.403, respectively. There were significant differences (P < 0.05) among the three groups in terms of duration of diabetes, previous PCI, previous stroke, BMI, SBP, DBP, HR, GRACE score, multi-vessel disease, treatment strategy, FBG, HbA1c, HDL-C, Uric acid, NT-proBNP, eGFR and the use of medications at discharge including clopidogrel or ticagrelor, β-blocker, angiotensin-converting enzyme inhibitor (ACE-I) or angiotensin II receptor blocker (ARB) and insulin, and no significant difference was found in the other indicators. The associations between the TyG index and cardiovascular risk factors were examined using linear regression analysis. As shown in Table 2, TyG index levels were positively associated with BMI, hemoglobin A1c (HbA1c) and uric acid and negatively associated with age, male sex, HDL-C and eGFR in the multivariate linear regression analysis (P < 0.05)

**Table 1:** Baseline characteristics of three groups

Variable	Tertile 1 (n = 844)	Tertile 2 (n = 843)	Tertile 3 (n = 844)	P value
TyG index	8.467 ± 0.293	9.114 ± 0.152	9.841 ± 0.403	< 0.001
Age, years	67.2 ± 6.9	66.2 ± 6.7	65.6 ± 6.8	< 0.001
Male	519 (61.5)	446 (52.9)	450 (53.3)	< 0.001
Duration of diabetes, years	10.2 ± 8.0	9.3 ± 7.3	10.0 ± 7.7	0.030
Newly diagnosed diabetes	47 (5.6)	53 (6.3)	57 (6.8)	0.596
Smoker	338 (40.0)	323 (38.3)	338 (40.4)	0.703
Hypertension	627 (74.3)	656 (77.8)	661 (78.3)	0.102
Family history	101 (12.0)	93 (11.0)	77 (9.1)	0.157
Previous MI	119 (14.1)	95 (11.3)	94 (11.1)	0.110
Previous PCI	193 (22.9)	156 (18.5)	149 (17.7)	0.015
Previous CABG	44 (5.2)	29 (3.4)	29 (3.4)	0.101
Previous stroke	138 (16.4)	187 (22.2)	190 (22.5)	0.002
BMI, kg/m^2^	25.4 ± 2.7	25.9 ± 2.7	26.4 ± 3.3	< 0.001
SBP, mmHg	134.9 ± 11.8	135.8 ± 12.0	136.9 ± 11.4	0.003
DBP, mmHg	74.0 ± 10.4	74.8 ± 10.6	75.9 ± 10.1	0.001
HR, bpm	72.9 ± 12.2	73.6 ± 12.1	74.9 ± 11.5	0.003
LVEF	58 ± 8	58 ± 8	57 ± 9	0.294
GRACE score	135 (129–140)	135 (130–141)	136 (131–142)	< 0.001
Clinical presentation				0.236
UAP	692 (82.0)	672 (79.7)	654 (77.5)	
NSTEMI	73 (8.6)	86 (10.2)	91 (10.8)	
STEMI	79 (9.4)	85 (10.1)	99 (11.7)	
Left main disease	82 (9.7)	94 (11.2)	87 (10.3)	0.624
Multi-vessel disease	658 (78.0)	689 (81.7)	697 (82.5)	0.037
Treatment strategy				0.001
Medicine therapy	310 (36.7)	249 (29.5)	241 (28.6)	
PCI	436 (51.7)	497 (59.0)	514 (60.9)	
CABG	98 (11.6)	97 (11.5)	89 (10.5)	
Laboratory findings				
Hemoglobin, g/dl	132.1 ± 15.8	133.0 ± 15.4	132.5 ± 15.3	0.490
FBG, mmol/L,	7.7 ± 2.8	7.9 ± 2.8	8.4 ± 3.4	<0.001
HbA1c, %	7.5 ± 1.4	7.6 ± 1.4	7.8 ± 1.4	<0.001
TC, mmol/L	4.40 ± 1.21	4.46 ± 1.06	4.39 ± 1.10	0.367
TG, mmol/L	1.50 (1.11–2.04)	1.53 (1.12–2.07)	1.54 (1.11–2.15)	0.699
LDL-C, mmol/L	2.88 ± 1.05	2.92 ± 0.93	2.90 ± 0.95	0.759
HDL-C, mmol/L	1.10 ± 0.32	1.08 ± 0.29	1.02 ± 0.28	< 0.001
Uric acid, umol/L	305.4 ± 78.4	306.4 ± 88.2	325.5 ± 106.1	< 0.001
hs-CRP, mg/L	1.89 (0.83–4.61)	1.64 (0.71–4.78)	1.85 (0.79–4.63)	0.325
NT-proBNP, pg/ml	108.1 (49.7–278.6)	117.8 (85.6–170.7)	160.8 (95.8–363.2)	< 0.001
eGFR, mL/min	97.8 ± 20.8	96.2 ± 23.7	85.5 ± 24.6	< 0.001
Medications at discharge				
Aspirin	817 (96.8)	811 (96.2)	814 (96.4)	0.799
Clopidogrel/Ticagrelor	666 (78.9)	689 (81.7)	707 (83.8)	0.036
β-blocker	513 (60.8)	545 (64.7)	586 (69.4)	0.001
ACEI/ARB	455 (53.9)	490 (58.1)	500 (59.2)	0.066
Statin	802 (95.0)	807 (95.7)	797 (94.4)	0.468
CCB	241 (28.6)	253 (30.0)	231 (27.4)	0.485
Nitrate	478 (56.6)	459 (54.4)	453 (53.7)	0.447
Insulin	321 (38.0)	327 (38.8)	377 (44.7)	0.010

Data are expressed as mean ± SD, medians with interquartile ranges or percentage

*TyG index* triglyceride-glucose index, *MI* myocardial infarction, *PCI* percutaneous coronary intervention, *CABG* coronary artery bypass graft, *BMI* body mass index, *SBP* systolic blood pressure, *DBP* diastolic blood pressure, *HR* heart rate, *LVEF* left ventricle ejection fraction, *GRACE Score* Global Registry of Acute Coronary Events Score, *UAP* unstable angina pectoris, *NSTEMI* non-ST-segment elevation myocardial infarction, *STEMI* ST-segment elevation myocardial infarction, *FBG* fasting blood glucose, *HbA1c* Hemoglobin A1c, *TC* total cholesterol*, TG* triglycerides*, LDL*-*C* low-density lipoprotein cholesterol, *HDL*-*C* high-density lipoprotein cholesterol, *hs*-*CRP* high-sensitivity C-reactive protein, *NT*-*proBNP* N-terminal proB-type natriuretic peptide, *eGFR* estimated glomerular filtration rate, *ACEI* angiotensin II coenzyme inhibitor, *ARB* angiotensin II receptor blocker, *CCB* calcium channel blocker, *SD* standard deviation

**Table 2:** Univariate and multivariate liner regression analysis for TyG index

Variable	Univariate			Multivariate		
	β	Standard β	P value	β	Standard β	P value
Age	− 0.11	− 0.114	< 0.001	− 0.010	− 0.106	< 0.001
Male	− 0.096	− 0.075	< 0.001	− 0.114	− 0.089	< 0.001
Smoker	0.019	0.015	0.454			
Hypertension	0.016	0.010	0.601			
BMI	0.051	0.235	< 0.001	0.046	0.209	< 0.001
HbA1c	0.041	0.090	< 0.001	0.030	0.065	0.001
LDL-C	0.001	0.002	0.914			
HDL-C	− 0.298	− 0.138	< 0.001	− 0.202	− 0.094	< 0.001
Uric acid	0.001	0.212	< 0.001	0.001	0.088	< 0.001
hs-CRP	0.001	0.027	0.182			
eGFR	− 0.008	− 0.295	< 0.001	− 0.006	− 0.226	< 0.001

*BMI* body mass index, *HbA1c* Hemoglobin A1c, *LDL*-*C* low-density lipoprotein cholesterol, *HDL*-*C* high-density lipoprotein cholesterol, *hs*-*CRP* high-sensitivity C-reactive protein, *eGFR* estimated glomerular filtration rate

### TyG index and cardiovascular events

**Fig. 1:**
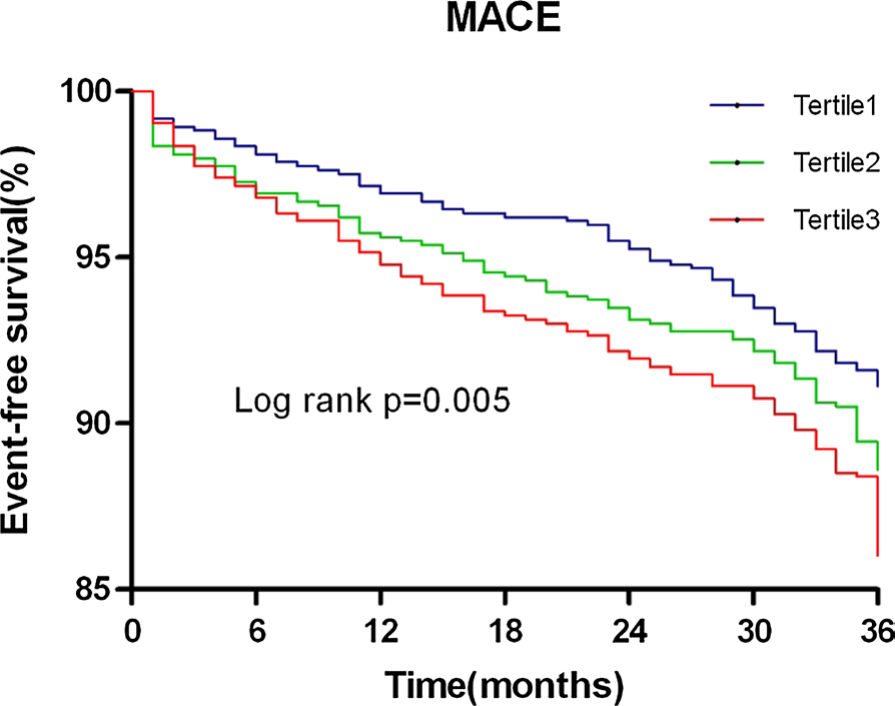
Kaplan–Meier survival curve for MACE (major adverse cardiovascular events) across TyG index tertiles

During the 3-year follow-up, 289 (11.4%) MACEs were recorded, including 142 (49.1%) all-cause death, 101 (34.9%) non-fatal MI and 46 (16.0%) non-fatal stroke. Table 3 shows the 3-year event rate and Cox proportional hazard analysis for all-cause death, non-fatal MI, non-fatal stroke and MACE. Rates of all-cause death, non-fatal MI, non-fatal stroke and MACE increased progressively with a higher TyG index. On unadjusted Cox modeling, only the rate of MACE rose significantly with elevated TyG index levels (P = 0.005 for trend). Multivariate-adjusted hazard ratio (HR) also increased with rising TyG index levels after adjusting for age, male, smoking history, previous MI, previous CABG, BMI, AMI, LVEF, left main disease, multi-vessel disease, HbA1c, hs-CRP, statin use and insulin use (P = 0.019 for the trend). As shown in Fig. 1, Kaplan–Meier survival analysis showed that the cumulative incidence of MACE increased with higher tertiles of the TyG index (log-rank test, P = 0.005).

**Table 3:** Baseline TyG index and Prediction of Cardiovascular Events

End point	Baseline TyG index	Events, n/total	3-year event rate, %	Unadjusted HR (95% CI)	P for trend	Adjusted HR (95% CI)	P for trend
All-cause death	Tertile 1	41/844	4.86	Ref.	0.238	Ref.	0.518
	Tertile 2	45/843	5.34	1.114 (0.730–1.701)		1.071 (0.694–1.651)	
	Tertile 3	56/844	6.64	1.397 (0.934–2.091)		1.266 (0.828–1.936)	
Non–fatal MI	Tertile 1	23/844	2.73	Ref.	0.067	Ref.	0.117
	Tertile 2	38/843	4.51	1.680 (1.001–2.819)		1.591 (0.939–2.697)	
	Tertile 3	40/844	4.74	1.782 (1.067–2.976)		1.709 (1.006–2.903)	
Non-fatal stroke	Tertile 1	11/844	1.30	Ref.	0.101	Ref.	0.115
	Tertile 2	13/843	1.54	1.200 (0.538–2.679)		1.237 (0.550–2.781)	
	Tertile 3	22/844	2.61	2.050 (0.994–4.227)		2.065 (0.983–4.341)	
MACE	Tertile 1	75/844	8.89	Ref.	0.005	Ref.	0.019
	Tertile 2	96/843	11.39	1.300 (0.961–1.758)		1.267 (0.932–1.723)	
	Tertile 3	118/844	13.98	1.611 (1.206–2.152)		1.537 (1.138–2.076)	

Adjusted variables were age, male, smoker, previous MI, previous CABG, BMI, AMI, LVEF, left main disease, multi-vessel disease, HbA1c, hs-CRP, statin, insulin

*MI* myocardial infarction, *MACE* major adverse cardiovascular event, *HR* hazard ratio, *CI* confidential interval

Univariate and multivariate Cox proportional hazards regression analyses and predictors for MACE are presented in Table 4. In the univariate analysis, the criteria associated with MACE occurrence were TyG index, age, previous MI, BMI, AMI, LVEF, left main disease, multi-vessel disease, hs-CRP and statin use. After adjusting for BMI and other confounding factors, multivariate Cox proportional hazards regression analysis showed that TyG index, age, previous MI, LVEF, hs-CRP and statin use independently predicted the occurrence of MACE in patients with diabetes and ACS.

**Table 4:** Univariate and multivariate Cox regression analysis for predicting MACE

Variables	HR	Univariate	P value	HR	Multivariate	P value
		95% CI			95% CI	
TyG index	1.471	1.238–1.748	< 0.001	1.455	1.208–1.753	< 0.001
Age	1.041	1.024–1.058	< 0.001	1.039	1.022–1.057	< 0.001
Male	1.227	0.969–1.554	0.089			
Duration of diabetes	1.012	0.997–1.026	0.117			
Smoker	1.253	0.994–1.581	0.056			
Hypertension	0.965	0.736–1.265	0.796			
Previous MI	1.807	1.350–2.419	< 0.001	1.439	1.048–1.975	0.024
Previous PCI	1.221	0.928–1.607	0.154			
Previous CABG	1.842	1.170–2.901	0.008			
Previous stroke	0.991	0.745–1.319	0.951			
BMI	1.045	1.005–1.086	0.027			
AMI	1.939	1.514–2.484	< 0.001			
LVEF	0.955	0.945–0.966	< 0.001	0.968	0.955–0.981	< 0.001
Left main disease	1.600	1.161–2.206	0.004			
Multi-vessel disease	1.568	1.119–2.197	0.009			
Revascularization	0.873	0.677–1.125	0.294			
HbA1c	1.077	0.997–1.164	0.061			
LDL-C	1.085	0.966–1.218	0.171			
Uric acid	1.001	0.999–1.002	0.276			
hs-CRP	1.009	1.005–1.012	< 0.001	1.004	1.000–1.008	0.031
NT-proBNP	1.001	0.999–1.003	0.331			
eGFR	0.997	0.992–1.002	0.247			
Statin	0.599	0.388–0.926	0.021	0.578	0.371–0.901	0.015
Insulin	1.210	0.960–1.526	0.107			

*TyG index* triglyceride-glucose index, *MI* myocardial infarction, *PCI* percutaneous coronary intervention, *AMI* acute myocardial infarction, *LVEF* left ventricle ejection fraction, *HbA1c* Hemoglobin A1c, *LDL*-*C* low-density lipoprotein cholesterol, *hs*-*CRP* high-sensitivity C-reactive protein, *NT*-*proBNP* N-terminal proB-type natriuretic peptide, *eGFR* estimated glomerular filtration rate, *MACE* major adverse cardiovascular event, *HR* hazard ratio, *CI* confidential interval

The ROC analysis showed that the optimal cutoff value of the TyG index level for predicting MACE was 9.323 (sensitivity 46.0% and specificity 63.6%), with an area under the curve (AUC) of 0.560 (95% CI: 0.524–0.595, P = 0.001). The incremental predictive value of the TyG index for MACE is shown in Table 5. Adding the TyG index to the model of established risk factors improved the prediction of MACE (P = 0.01). Moreover, the addition of the TyG index has an incremental prognostic value for predicting MACE in terms of NRI (14.7% improvement, P = 0.019) and IDI (8.9% improvement, P = 0.001), especially when comparing the baseline model with established risk factors.

**Table 5:** Evaluation of Predictive Models for MACE

	C-Statistic	P value	NRI (95% CI)	P value	IDI (95% CI)	P value
Established risk factors	0.649 (0.613–0.686)	Ref.		Ref.		Ref.
Established risk factors + TyG index	0.677 (0.644–0.711)	0.010	0.147 (0.025–0.270)	0.019	0.090 (0.004–0.014)	0.001

*TyG index* triglyceride-glucose index, *MACE* major adverse cardiovascular event, *NRI* net reclassification improvement, *IDI* integrated discrimination improvement. Established risk factors included age, previous MI, LVEF, hs-CRP and statin

The prognostic values of the TyG index in various subgroups for MACE are presented in Table 6. After adjusting for age, sex, duration of diabetes, smoking status, hypertension, previous MI, previous PCI, previous CABG, previous stroke, BMI, LVEF, left main disease, multi-vessel disease, HbA1c, LDL-C, uric acid, hs-CRP, NT-proBNP, eGFR, statin use and insulin use, the TyG index still independently predicted the occurrence of MACE in patients with diabetes and ACS irrespective of treatment strategy. The TyG index independently predicted the occurrence of MACE in the UAP subgroup, while it could not independently predict the occurrence of MACE in NSTEMI and STEMI subgroups.

**Table 6:** Prognostic value of TyG index for MACE in various subgroups

Variables	TyG index					
	Medicine therapy	PCI	CABG	UAP	NSTEMI	STEMI
Adjusted HR (95% CI)	1.854 (1.262–2.723)	1.315 (1.014–1.705)	2.014 (1.093–3.708)	1.604 (1.270–2.027)	1.261 (0.754–2.109)	1.195 (0.639–2.235)
P value	0.002	0.039	0.025	<0.001	0.377	0.577

*TyG index* triglyceride-glucose index, *PCI* percutaneous coronary intervention, *CABG* coronary artery bypass graft, *UAP* unstable angina pectoris, *NSTEMI* non-ST-segment elevation myocardial infarction, *STEMI* ST-segment elevation myocardial infarction, *MACE* major adverse cardiovascular event, *HR* hazard ratio, *CI* confidential interval

## Discussion

This study investigated the association between the TyG index and MACE in patients with diabetes and ACS. The results showed the TyG index was positively associated with increased MACE. After adjusting for confounding factors, the TyG index was an independent predictor of MACE irrespective of treatment strategy. Furthermore, our results showed that adding the TyG index to the model may improve the discrimination of risk prediction for MACE in patients with diabetes and ACS. These findings revealed the prognostic value of the TyG index for MACE in patients with diabetes and ACS. To the best of our knowledge, this study demonstrated, for the first time, that the TyG index is a potential predictor for MACE in patients with diabetes and ACS who received different treatments. Most importantly, this study suggests that a simple method of estimating IR may optimize the risk stratification of recurrent cardiovascular risk in patients with diabetes and ACS.

IR is a major characteristic of T2DM and has been recognized as a risk factor for CVD [27]. IR not only contributes to the development of CVD in both the general population and patients with diabetes but also predicts cardiovascular outcomes in patients with CVD [28, 29]. Therefore, identification of IR will have great clinical significance for improving cardiovascular risk stratification in primary and secondary prevention. However, there is no consensus on whether IR predicts cardiovascular risks in patients with established diabetes, with or without CVD [30–32]. A recent study demonstrated that the degree of IR, reflected by HOMA-IR, was not associated with CVEs in patients with diabetes and ACS who are not treated with insulin [33]. The TyG index, as the product of FPG and triglycerides, is a novel index that has been suggested as a simple and reliable surrogate of IR and has been shown to be superior to HOMA-IR in predicting IR [7, 8]. Compared with the HOMA-IR, the TyG index does not require quantification of insulin and may apply to all of the patients treated with insulin. It is well established that an increased TyG index is associated with increased risks of T2DM and CVD [6, 20–24]. Moreover, the TyG index has been recognized as an independent predictor for the risk of CVEs in patients with CVD [16–19]. Atherosclerotic CVD is the most common cause of death in patients with diabetes. Therefore, it is necessary to determine whether the TyG index predicts future cardiovascular risk in patients with T2DM and ACS.

Whether the TyG index is able to predict cardiovascular outcomes in patients with established T2DM remains controversial. In a study of 3524 patients with T2DM, Su et al. found the TyG index was positively associated with CVEs, including MI, UAP, stroke, hospitalization for CAD, peripheral artery disease and cardiovascular-related death, suggesting that the TyG index may be a useful marker for predicting clinical outcomes and may provide an additional prognostic benefit compared with HbA1c [34]. Data from a study of 1282 patients with T2DM with new-onset, stable CAD during a 3846-person-year follow-up by Jin et al. found that the TyG index could predict cardiovascular outcomes defined by cardiovascular mortality, non-fatal MI, stroke, post-discharge revascularization and hospitalized UAP, and that the TyG index may have better prognostic value for CVEs than hemoglobin glycation indexes (HGIs) [19]. In addition, a study by Ma et al. of 776 patients with T2DM and ACS who underwent PCI showed that the TyG index was significantly associated with cardiovascular outcomes, including all-cause mortality, non-fatal stroke, non-fatal myocardial infarction and unplanned repeat revascularization [25]. Collectively, these findings suggested that an increase in the TyG index is strongly correlated with the development of CVD in patients with T2DM. However, contrary to these studies, several other studies failed to demonstrate any association between the TyG index and CVEs. Laura et al. demonstrated that the TyG index was not associated with a 10-year CVD risk defined by CAD, cerebrovascular disease and peripheral arterial disease in 258 participants with T2DM [20]. Cho et al. failed to find an independent association between the TyG index and CAD or obstructive CAD in 996 established patients with diabetes after adjusting for traditional cardiovascular risk factors [35]. Differences in participant selection, event definition, or research methods may have contributed to the disparity of these results. Of note, data focused on patients with diabetes and ACS who received different treatment strategies have been scarce.

To our knowledge, our study population represents the largest cohort of patients with diabetes and angiographically proven ACS in which the association between the TyG index and long-term MACE has been investigated. Moreover, our study is the first to take all-cause death, non-fatal MI and non-fatal stroke as the composite endpoint events. Compared with a recent study [25], our study included patients who received different treatments. Moreover, patients in our study had a higher percentage of stroke, thus denoting higher risk patients. However, since most of them had been diagnosed with T2DM and had received hypoglycemic treatment before this admission, their overall HbA1c and FPG values were not very high. Our study demonstrated that a higher TyG index was significantly associated with a higher risk of MACE. The higher risk of MACE persisted after adjusting for traditional cardiovascular risk factors, burden of comorbidities, disease severity and medications. We also found that after adjusting for important variables, the TyG index remained independently predictive of adverse cardiovascular outcomes irrespective of treatment strategy. Thus, our study supports previous studies showing an association between the TyG index and adverse CVEs in patients ACS. However, the prognostic value of the TyG index only applied to patients with UAP, which was different than what we observed in patients with a general diagnosis of ACS. This indicated that the prognostic value of the TyG index may not be applicable to the entire range of ACS, and that the presence of AMI may attenuate the association between TyG index and MACE in patients with diabetes and ACS. In this study, we identified the optimal TyG index cut-off for predicting MACE. We found the AUC of the optimal cut-off value of 9.456 was poor, suggesting that it is difficult to predict hard endpoint events based on the TyG index alone. However, by adding the TyG index into established risk factors of MACE, we found a significant improvement in risk prediction in terms of the C-statistic value, NRI and IDI. Although previous studies have shown that a higher TyG index is associated with worse cardiovascular outcomes, none have discriminated the incremental prognostic value of the TyG index in terms of hard clinical endpoints. Our results implied that the use of the TyG index may refine the risk stratification of cardiovascular risk. Routinely introducing the TyG index into clinical diagnostic models could more accurately identify patients with higher cardiovascular risk, thus enabling a more targeted treatment or prevention.

Although the TyG index independently predicted cardiovascular events in T2DM with or without cardiovascular events [34], the association between the TyG index and these individual events in patients with T2DM has not been well demonstrated. We did not observe a significant association of the TyG index with all-cause death, non-fatal MI or non-fatal stroke in either unadjusted or adjusted analysis. These results may suggest that once patients have established diabetes and ACS, the TyG index may not determine the future risk of death, MI or stroke. Another possible explanation is that concomitant secondary prevention measures may affect the impact of the TyG index in predicting death, MI or stroke. In addition, the relatively small number of cardiovascular events we observed in our sample made it difficult to conclude the relationship between TyG index and these individual events.

The exact mechanisms accounting for the association between the TyG index and MACE remain unclear. As a reliable marker of the severity of IR, proatherogenic properties of IR may partly account for the association [36, 37]. In this study, TyG index levels were positively associated with BMI, HbA1c and uric acid, and were negatively associated with HDL-C and eGFR, suggesting that the observed association between the TyG index and poor prognosis may be explained by the presence of cardiovascular risk factors. Consistent with previous studies [17, 38], the TyG index was positively associated with the severity of CAD, suggesting that a difference in the extent of coronary atherosclerosis may contribute to the graded TyG index-MACE relationship. Moreover, the TyG index has been strongly associated with coronary artery calcification progression [39]. In addition, the TyG index has been correlated with micro- and macrovascular damage, such as arterial stiffness, nephric microvascular damage, cardiac autonomic neuropathy and cerebrovascular disease [40–42], all of the conditions known to increase the risk of adverse CVEs. Nevertheless, in this study, patients with higher TyG indices were younger and less likely to have PCI history and prior MI. Therefore, more efforts must be made to elucidate the exact mechanism of the association between the TyG index and CVEs.

### Study limitations

This study has several potential limitations. First, as this was a single-center retrospective study, it is difficult to exclude influence from some unmeasured and residual confounding factors. The lack of any information about the presence of diabetes complications may have exaggerated the results of this study, with albuminuria being the most relevant complication predicting CV events. Second, the diagnosis of diabetes was made by the attending physician and only those with diabetic symptoms underwent the OGTT test. Therefore, some patients with diabetes may have remained unidentified. Third, FPG and triglyceride levels were only measured at the baseline. The levels of FPG and triglyceride might have changed by the follow-up; therefore, it is unknown whether the change in the TyG index could have predicted cardiovascular outcomes. Fourth, the Synergy Between PCI With Taxus and Cardiac Surgery (SYNTAX) score was not routinely calculated in our cardiac catheterization lab, so the association between the TyG index and the SYNTAX score was not evaluated. Therefore, future studies should explore the impact of the TyG index on the SYNTAX score in patients with diabetes and ACS. Our research also did not include the HOMA-IR index. Further study on the comparison of the predictive value of the TyG index and HOMA-IR must be explored. We also did not compare the predictive value of the TyG index and HbA1c because the predictive value of the TyG index only remained significant when the two variables were in the same multivariate Cox regression model. Fifth, the study was based on Chinese patients; therefore, these results require replications in other ethnic cohorts. Finally, although our study did not demonstrate the prognostic value of the TyG index in patients with diabetes and AMI, this finding requires further evaluation in a larger, prospective study. Despite these limitations, this study has important clinical implications because it is the first to investigate the association between the TyG index and MACE in patients with established diabetes and ACS who received different treatments.

## Conclusion

A high TyG index was independently associated with an increased risk of MACE in patients with diabetes and ACS. Adding the TyG index to the basic model provided has an incremental prognostic value for the prediction of MACE. These findings suggested that the TyG index may be a useful marker for risk stratification and prognosis in patients with diabetes and ACS who have received different ACS treatments.

## Data Availability

The datasets used and/or analyzed during the current study are available from the corresponding author on reasonable request.

## Abbreviations

CAD: Coronary artery disease
ACS: Acute coronary syndrome
CVEs: Cardiovascular events
TyG index: Triglyceride-glucose index
IR: Insulin resistance
HOMA-IR: Homeostasis model assessment of IR
CVD: Cardiovascular disease
T2DM: Type 2 diabetes mellitus
FBG: Fasting blood glucose
UAP: Unstable angina pectoris
NSTEMI: Non-ST-segment elevation myocardial infarction
STEMI: ST-segment elevation myocardial infarction
MI: Myocardial infarction
PCI: Percutaneous coronary intervention
CABG: Coronary artery bypass graft
SBP: Systolic blood pressure
DBP: Diastolic blood pressure
HR: Heart rate
LVEF: Left ventricle eject fraction
HbA1c: Hemoglobin A1c
TC: Total cholesterol
TG: Triglyceride
LDL-C: Low density lipoprotein-C
HDL-C: High density lipoprotein-C
hs-CRP: High-sensitivity C-reactive protein
NT-proBNP: N-terminal proB-type natriuretic peptide
eGFR: Estimated glomerular filtration rate
BMI: Body mass index
MACE: Major adverse cardiovascular event
NRI: Net reclassification improvement
IDI: Integrated discrimination improvement
ROC: Receiver operating characteristic
AUCs: Area under the receiver operating characteristic curves
SYNTAX: The Synergy Between PCI With Taxus and Cardiac Surgery
